# Supplementary material for: Splice-Junction-Based Mapping of Alternative Isoforms in the Human Proteome
Source: Cell Rep. Author manuscript; Available in PMC 2020 Jan 15. (PMC6961840; doi:10.1016/j.celrep.2019.11.026)
Supplement: 2 [file NIHMS1546469-supplement-2.zip › DF1/PXD010154/Lung/Lung_24_GAK_GVRAPSFGSPAGFPPGGFIPK.pdf]

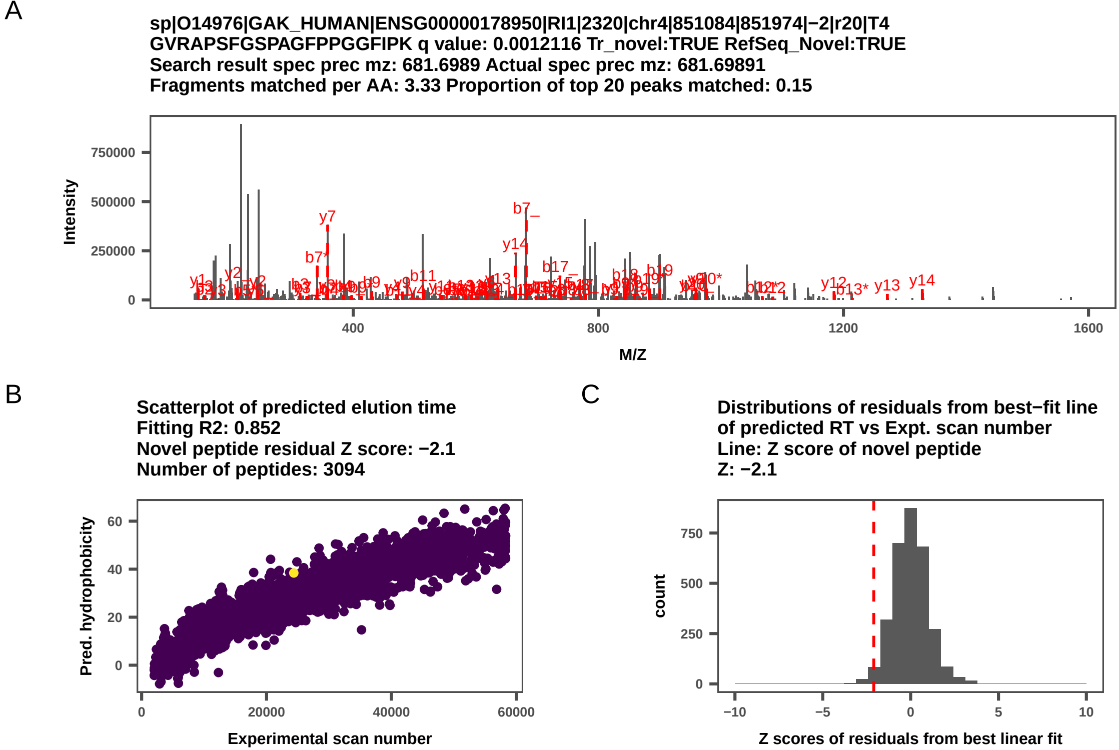
A
sp|O14976|GAK_HUMAN|ENSG00000178950|RI1|2320|chr4|851084|851974|−2|r20|T4
GVRAPSFGSPAGFPPGGFIPK q value: 0.0012116 Tr_novel:TRUE RefSeq_Novel:TRUE
Search result spec prec mz: 681.6989 Actual spec prec mz: 681.69891
Fragments matched per AA: 3.33 Proportion of top 20 peaks matched: 0.15
Intensity
750000
500000
250000
0
M/Z
400
800
1200
1600
y7
b7*
b7_
y14
b17_
b11
b19
y12
b13*
y13
y14
B
Scatterplot of predicted elution time
Fitting R2: 0.852
Novel peptide residual Z score: −2.1
Number of peptides: 3094
Pred. hydrophobicity
60
40
20
0
0
20000
40000
60000
Experimental scan number
C
Distributions of residuals from best−fit line
of predicted RT vs Expt. scan number
Line: Z score of novel peptide
Z: −2.1
count
750
500
250
0
−10
−5
0
5
10
Z scores of residuals from best linear fit
